# Supplementary material for: Modelling interference between vectors of non-persistently transmitted plant viruses to identify effective control strategies
Source: PLoS Comput Biol. 2021 Dec 28;17(12):e1009727. doi: 10.1371/journal.pcbi.1009727 (PMC8758101; doi:10.1371/journal.pcbi.1009727)
Supplement: S3 Text — (PDF) [file pcbi.1009727.s003.pdf]

S3 Text. Agricultural practices and disease control

The responses of the incidence of virus infection in plants ($\bar{I}$) and of the basic reproduction number (R_0) to variation of plant hosting capacity (h), aphid mortality (μ) and infected plant roguing rate (ρ) are reported in the figure below. Details on the involved processes are given in the main text.

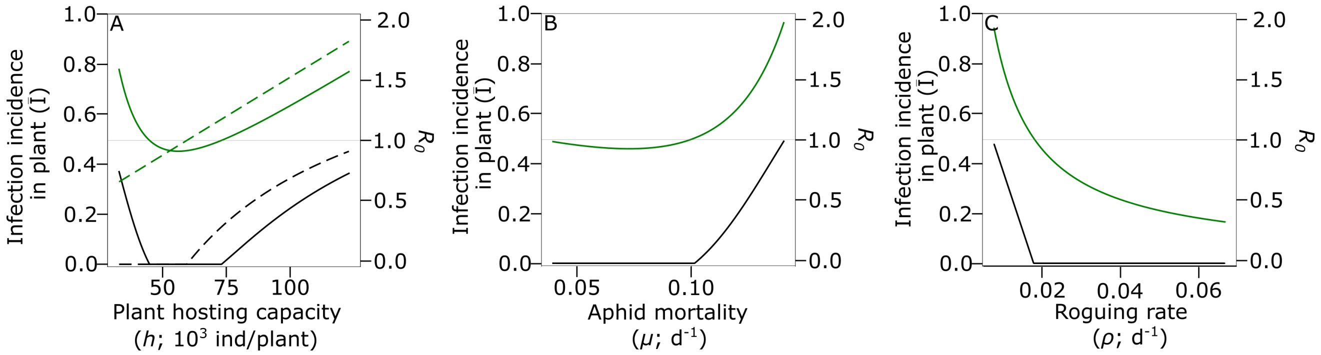

Figure S1: Response of the incidence of virus infection in plant at the equilibrium ($\bar{I}$, in black) and of the reproduction number (R_0, in green) to changes in (A) aphids hosting capacity (h) under indirect (continuous lines) and direct (dashed lines) interference scenarios; (B) resident aphids mortality (μ); (C) roguing rate (ρ).
