# Supplementary material for: Modelling interference between vectors of non-persistently transmitted plant viruses to identify effective control strategies
Source: PLoS Comput Biol. 2021 Dec 28;17(12):e1009727. doi: 10.1371/journal.pcbi.1009727 (PMC8758101; doi:10.1371/journal.pcbi.1009727)
Supplement: Attachment — Submitted filename: Final Response Letter.pdf [file pcbi.1009727.s005.pdf]

Dr Nik Cunniffe
Associate Professor
Department of Plant Sciences
University of Cambridge
Downing Street
Cambridge, CB2 3EA

UNIVERSITY OF CAMBRIDGE

Department of Plant Sciences

25 November, 2021

Manuscript PCOMPBIOL-D-21-01416

The role of interference between vectors in control of plant diseases

Dear Konstantin B. Blyuss and Virginia Pitzer,

Thank you for handling our manuscript, and for securing such useful reviews.

Please find below our responses to the reviewers' comments (note that the line numbering refers to the version of the manuscript in which additions and deletions are highlighted).

Note that the title as given directly above is for the manuscript as it was originally submitted. As part of our revision we have followed the suggestion of Reviewer #1, and have altered the title to *"Modelling interference between vectors of non-persistently transmitted plant viruses to identify effective control strategies"*. The intent is to emphasize that our paper is a modelling study of non-persistently transmitted plant viruses.

Thank you again for handling our manuscript.

Nik Cunniffe

(on behalf of all co-authors)

Telephone: +44 1223 333954
Fax: +44 1223 333953
E-mail: njc1001@cam.ac.uk

Reviewer #1

1) Plant viruses are major constraints to crop production worldwide. Most plant viruses are transmitted by insect or arthropod vectors, with several different transmission mechanisms. These transmission mechanisms lead to virus groupings that are not based on virus taxonomy, per se, but on epidemiologically relevant traits (time to inoculate a plant, time for vector to acquire the virus from a plant, infectious period in the vector, transmission to insect offspring, and so on). Nonpersistently transmitted (NPT) viruses comprise a large group with many economically and ecologically important viruses, many of which are transmitted by aphids (which can live/reside within the crop or are transitory, passing through the crop rather quickly while also transmitting a virus). There can be interactions between the resident and transient vector populations (direct and indirect), especially interference of transients by the residents, the subjects of this manuscript.
Although there have been several strong theoretical studies on modeling plant virus disease epidemics (properly cited in this manuscript), this is the first major theoretical modeling effort aimed at the impact of interference of vectors on the plant virus epidemics. Building on past theoretical and empirical work, the authors developed an extended deterministic SI coupled differential equation type model for plant viruses and their vectors. They define the many parameters and variables, and explore the impacts of vector interference AND some cropping systems conditions (fertilization, irrigation) on the plant epidemics. Importantly, they derived R0 for a subset of the conditions covered by the SI model. Although the model is (necessarily?) quite complex, the components are explained and justified, and they appear to be based on the current knowledge of the epidemiology of NPT plant viruses. The authors show the situations that lead to epidemics and no epidemics based on R0 (and the parameters that make up R0).

The manuscript does not present any empirical data, and I doubt that the SI model could ever be fit directly to data without pre-assigning most of the parameters to fixed values (or exploring different [highly] informative priors for them using Bayesian methods). However, I think there are sufficient interesting and illuminating theoretical results to justify acceptance. I found the results very interesting. These modeling results can inspire future field research. Results, as expected, show that there may a complex relation between epidemiological (or agronomic) parameters and epidemic outcomes. Vector interference is a epidemiological process that needs to be considered for developing control strategies for plant viruses with NPT vectors.

Although I feel the manuscript is valuable and warrants acceptance, I think the authors should consider a few items in their revision, as explained below. These are mostly for the Discussion.

Response. *It is a real pleasure for us reading that the reviewer appreciated our work. We thank him/her for his/her careful revision and for the observations and suggestions provided, as well as for the strong and unambiguous support for publication. As described in more detail below, we have consequently re-edited our manuscript, namely the discussion section, in response to all the reviewer's observations/suggestions.*

Action. *None requested.*

2) Title: Since this manuscript is for NPT viruses only, I think the title should reflect this. (The epidemiological properties of semi-persistently and persistently transmitted plant viruses are different). I also think that the title should reflect that this is a theoretical study.

Response. *We followed Reviewer 1's suggestion and we have provided a new title which focuses more tightly on NPT viruses, as well as makes it clear this is a modelling study.*

Action. *Title altered: "Modelling interference between vectors of non-persistently transmitted plant viruses to identify effective control strategies".*

3) The f(.) and g(.) equations 4 and 5 should have greater justification or explanation in the main body of the text (not just the supplement). I would like greater justification for the fixed parameters that are chosen, and a better explanation of the shape of the curves that are produced.

Response. *In the new version of the ms we reworded the text in "Modelling interference between resident and transient aphids" and "Visiting interference" sections, with the aim to make it clearer and so provide the greater explanation requested by Reviewer 1. Also, we provided a new figure 2 reporting the shape of the f(.) curve for the parameter values actually used for numerical simulations.*

In our theoretical work we wanted to stress the fact that an increase in the abundance of resident vectors i) decreases the number of visited plants by transient vectors and ii) increases their emigration rate from the system. It is thus crucial to have chosen monotonic functions, but the precise functional forms and values of parameters are not particularly important to set the responses we see. We explored the influence of the parameters of the interference functions (ν and α) on the response of R_0 and its components R_0^T and R_0^R to variation of plant hosting capacity (h), aphid mortality (μ) and infected plant roguing rate (ρ). The results presented in figure 3 in the main text are qualitatively unaffected by the values of ν and α (as showed, respectively, in the new figures S3 and S4 in the Supporting Information).

Precise values for parameters ν and α might be determined by sound experimental works for specific pathosystems if one were interested in finding the thresholds values of resident vector densities. On the other hand, in our work we are interested in showing the qualitative behaviour of the system.

Action. *We reworded the text in "Modelling interference between resident and transient aphids" and "Visiting interference" sections* (L187-L192, L195, L199-L200, L208-L211). *We provided a new figure 2 reporting the shape of the f(.) curve for the parameter values actually used for numerical simulations (and we changed the caption accordingly).*

We stated in the Discussion that the results we show in figure 3 are qualitatively unaffected by the values of parameters ν and α of the interference functions (L389-L393).

In the Supporting Information, we explored the effect of three values of ν and of three values of α on the response of R_0 and its components R_0^T and R_0^R to variation of plant hosting capacity (h), aphid mortality (μ) and infected plant roguing rate (ρ) (L142-L164 and figures S3 and S4 in the Supporting Information).

4) The authors clearly explain that their derived R0 (eqn 6) is only for the situation when there are no immigrant viruliferous (infectious) vectors (pi=0). In fact, I think that R0 may not be definable here is pi>0), although I would have to think further about that. The authors nicely ex and plore the effects of many important epidemiological parameters on R0 in this manuscript. But what can we conclude about the situation (and especially the role of interference) when pi>0 in terms of plant virus epidemics? A not uncommon scenario is for immigrant vectors to be carrying the virus (say, from surrounding infected weeds or other neighboring crops). Many years ago, Mike Irwin studied this in the field with soybean NPT viruses. This is probably a new theoretical investigation for the future, but the authors should certainly address this more in the Discussion.

Response. *The reviewer is right, the reason why we focused our numerical analysis on the case $\pi = 0$ is because only in this case can R_0 be properly defined.*

The textbook definition of R_0 – "the average number of secondary cases arising from an average primary case, in an entirely susceptible population" [Keeling & Rohani (2008) Modelling Infectious Disease in Humans and Animals] – is rather difficult to motivate when there is also immigration of infected individuals. Certainly any value that was extracted from a model would lose its normal meaning as a threshold for disease spread – or its interpretation as the exponential rate of increase in the number of infections per generation [Diekmann et al. (1990) J. Math. Biol. 28(4):365–382] – in this case.

The simple definition above can be used to motivate a number of different methods of calculating R0, each of which can give different results, [Li et al (2011) Computational and Mathematical Methods in Medicine. 2011:527610]. For at least the last decade, operational definitions have been based on the so-called Next Generation Matrix, which allows relatively simple calculation of R_0 in a way that properly accounts for various subtleties due to heterogeneity [Diekmann et al. (2010) J. R. Soc. Interface. 7:873-885]. The Next Generation Matrix requires as a first step the linearization of the system around its disease-free-equilibrium. As shown in our mathematical analysis, in the case for $\pi > 0$, the disease is always able to persist. Since there is no disease-free-equilibrium, most disease epidemiologists would therefore consider it is simply not possible to define R_0.

Action. *We better explained the methodology used to compute R_0 and its implications in the Results (L271-272 and L277-285) and in the Discussion, along with the need for further investigation on the effect of immigration of infectious vectors on the presented results (L438-452).*

5) The authors have taken a strictly SI modeling approach. Although this is adequate for their objectives (in my view), they should discuss in the Discussion some of the implications of this simplifying modeling choice (of course, the model is complex in other ways). For instance, they ignore the latent (E, exposed) and removed (R) diseased states in plants. This does create a more artificial situation (or very specific) epidemiological situation than found in general for plant epidemics (although a common simplifying SEIR modeling tool). Ignoring E (i.e., assuming that the latent period is 0) won't effect R0, or have little effect, but would affect the rate of disease increase in the model. A removed state is indirectly achieved by roguing or harvesting.

However, plants _can_ naturally move from the I to the R states without human activity, although the infectious period in the plant may be long enough that little of this transition occurs (for some diseases for some crops, such as for annuals). But this all deserves some attention in the Discussion. That is, what conclusions could be affected by use of SI rather than SEIR plant model? I realize that an SI type model is also used for the two vector populations, but this makes perfectly sense for NPT plant viruses (no latent period in the vector and aphids lose the plant virus very quickly and become virus-free again).

Response. *We agree with the reviewer comment. Thanks to this comment we deeper explored the consequences of our modelling choices, that are now presented in the Discussion.*

Action. *We added a new paragraph in the Discussion (L415-435)*

6) The authors put a big emphasis on roguing (removing of diseased plants). This makes sense for some crops, such as trees or high-value ornamentals. However, roguing is never (or rarely) done for many other crops, such as arable or field crops (maize, wheat, soybeans), or annuals in general. Moreover, roguing only sometimes is done in conjunction with replacement with a new disease-free plant (what the model is assuming). In many cases, diseased trees are simply removed without replacement. Based on the authors' SI model, these latter scenarios (leading to a nonconstant total plant host population) are not considered. Of course, all models are simplifications, but the authors should address their simplifications in their Discussion (i.e., future needed work).

Response. *Yes, we agree we could be more expansive about our assumption on roguing. The reviewer is right and we decided to address this simplification in the discussion.*

Action. *We addressed this simplification in the discussion (see L512-516)*

Reviewer #2

1) This paper theoretically explores the effects of interference competition between two vector species on the spread of a plant virus. One vector species is resident and the other is transient on the host plant. Only the resident species is vulnerable to insecticides. The resident species can repel the transient species either directly (competition for space) or indirectly (by the emission of volatiles compounds by the plant). Technically, the authors consider a SIR-like ODE system composed of 6 six equations. The originality of their work is to consider two vector species and their interferences through functions that depend negatively on the (relative) number of resident vectors per plant to describe direct (respectively indirect) interference mechanisms. Both visitation and emigration interference are considered. The authors show that increasing irrigation and/or fertilization, and therefore increasing the host capacity of the plant, has a unimodal effect on disease incidence or its proxy (the basic reproductive number R0) when interference is indirect. Specifically, a moderate use of irrigation and/or fertilization may maximize interference between vectors and therefor minimize disease incidence (Fig. 3A). Similarly, a minimum value of R0 is obtained for intermediate values of pesticide-induced resident mortality (Fig. 3B). Interestingly, the optimal pesticide application rate is positively

related with the irrigation/fertilization rate (Fig. 4A). This means that eradication of the disease can be achieved either by high irrigation / fertilization and high use of pesticides, or by low irrigation / fertilization and low use of pesticides.

Response. *We thank the reviewer for the time spent revising our work: we are glad that s/he appreciated it and we are thankful for the comments provided.*

Action. *None (no changes requested/suggested by referee in the above comment).*

2) The manuscript is very clear and well written. I spotted only one typo: line 337, summarizeD. The mathematical analysis of the model is clear and rigorous.

Response. *We thank the reviewer for noting it.*

Action. *Corrected typo (L357).*

3) I have only one remark: in the abstract, the authors refer to pesticides promoting the spread of NPT viruses as a counter-intuitive result, while it is a direct consequence of a model assumption. Namely, pesticides only kill resident vectors, not transient vectors. Since the authors refer to this lack of effect of pesticides on transient vectors as being well known, and since transient vectors are the main driver of disease spread, the result is rather expected. I suggest deleting "counter-intuitively" in the abstract.

Response. *We originally wrote it because farmers deploy chemicals against aphids imagining to control viral diseases, giving that also resident aphids can spread NPT viruses. Our results are then counter-intuitive in that sense. Moreover, there is an optimal value of pesticide induced mortality (μ) that minimizes R0, which is not a priori expected given our assumptions. For all these reasons, we have chosen to keep the term counter-intuitively in the abstract. However, if the editor prefers, we could certainly make a change in response to this comment.*

Action. *Currently no change (we will happily make the change to the abstract if the Editor prefers).*

4) By the way, since it is also well know that pesticides can kill non-target species, I would like to have more explanations of why transient vectors are spared from pesticides in the Discussion.

Response. *Thank you for this comment that allows us to better explain the effect of pesticides on transient vectors*

In the paper, we did not aim to distinguish the effect of pesticide on target vs non-target species, but the effect of the pesticides on species that spend sufficiently time within the considered system to be sensitive to the pesticides. Transient aphids rapidly pass through the field, landing on plant and doing epidermic exploratory probing. After such exploratory probing, they quickly disperse to another host, having rejected the plant as unpalatable. Thus, since transient aphids rapidly passed through the field without colonizing any plant, it is rare that they are exposed to a lethal dose of pesticides before leaving the field.

Action. *We better explained in the Discussion why the transient vectors are not sensible to the effect of pesticides (see L474-485).*

Reviewer #3

A good modelling study

Response. *We are glad the reviewer appreciated our work.*

Action. *None (no changes requested/suggested by referee in the above comment).*
